# Motile *Onchocerca volvulus* Microfilariae in the Anterior Chamber of the Eye

**DOI:** 10.4269/ajtmh.20-0025

**Published:** 2020-05

**Authors:** Michael E. Gyasi, Augustine R. Hong, Gary J. Weil

**Affiliations:** 1 St. Thomas Eye Hospital, Accra, Ghana;; 2 Department of Ophthalmology, Washington University School of Medicine, St. Louis, Missouri;; 3 Infectious Diseases Division, Department of Medicine, Washington University School of Medicine, St. Louis, Missouri

A 59-year-old man from an onchocerciasis-endemic area in eastern Ghana was enrolled in a clinical trial to assess the impact of ivermectin on microfilariae (Mf) in the skin and eyes. He had palpable subcutaneous nodules consistent with onchocerciasis and a high Mf burden in skin snips (170/mg of skin). He reported mild generalized pruritus and blurry vision in both eyes. Visual acuity was 20/80 (OD) and 20/60 (OS). Both eyes had moderate cataracts and features consistent with prior anterior uveitis. Intraocular pressures were normal (12 mmHg OU). Atrophic changes in his optic nerves correlated well with visual field defects measured by frequency doubling technology and with retinal nerve fiber layer loss as assessed by optical coherence tomography. Slit lamp examination revealed high numbers of motile Mf in the anterior chambers of both eyes (100-OD and 50-OS) (Figure 1). A cell-phone video (Supplemental Video) illustrates the dramatic appearance of Mf in the anterior chamber by a slit lamp.

**Figure 1.**
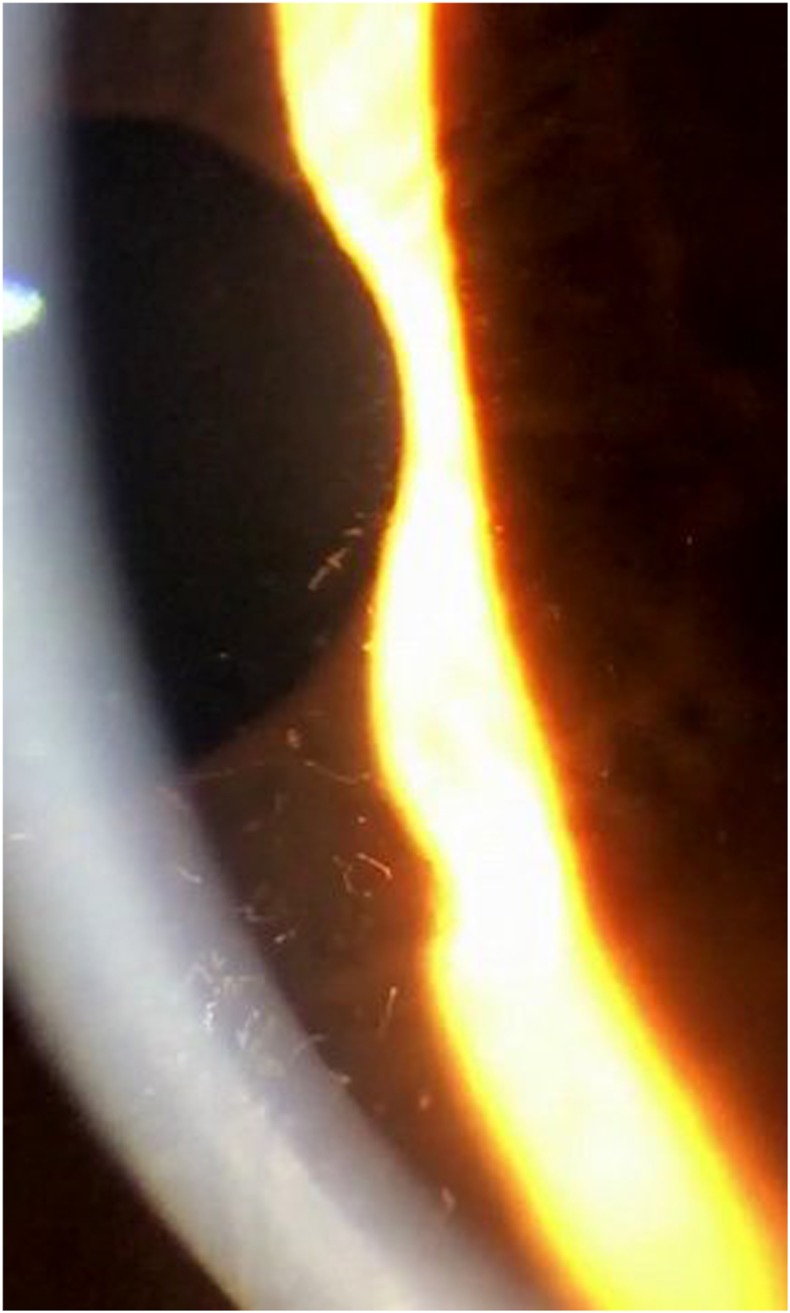
This screenshot from the slit lamp video shows *Onchocerca volvulus* microfilariae (Mf) in the anterior chamber of the eye. Some Mf overlie the pupil (in the upper left of the image). This figure appears in color at www.ajtmh.org.

Ocular Mf were still present but decreased in numbers and motility (40-OD and 20-OS) 3 months after ivermectin treatment (150 mg/kg); no Mf were detected in four skin snips taken from each iliac crest and calf at that time. Microfilariae were still absent in skin snips taken 6 months after treatment when ocular Mf counts were 20-OD and 15-OS.

*Onchocerca volvulus* Mf were cleared from the eyes more slowly than from the skin after ivermectin treatment. The patient’s vision did not improve after treatment presumably because of irreversible effects of onchocerciasis.

## Supplemental video

Supplemental materials

