# Supplementary material for: Motile Onchocerca volvulus Microfilariae in the Anterior Chamber of the Eye
Source: Am J Trop Med Hyg. 2020 May;102(5):921. doi: 10.4269/ajtmh.20-0025 (PMC7204589; doi:10.4269/ajtmh.20-0025)
Supplement: Supplementary file 2 [file tpmd200025.SD2.pdf]

Supplemental Video

Supplementary material (video). This pre-treatment slit lamp video shows many motile *Onchocerca volvulus* microfilariae in the anterior chamber of the eye.

Note: Supplemental Video will appear online in final publication
